# On the Question of Sporadic or Atypical Bovine Spongiform Encephalopathy and Creutzfeldt-Jakob Disease

**DOI:** 10.3201/eid1212.060965

**Published:** 2006-12

**Authors:** Paul Brown, Lisa M. McShane, Gianluigi Zanusso, Linda Detwiler

**Affiliations:** * Bethesda, Maryland, USA;; † National Institutes of Health, Bethesda, Maryland, USA;; ‡ University of Verona, Verona, Italy,; § Virginia-Maryland Regional College of Veterinary Medicine, College Park, Maryland, USA

**Keywords:** Bovine spongiform encephalopathy, bovine amyloid spongiform encephalopathy (BASE), Creutzfeldt-Jakob disease, diagnostic screening tests, perspective

## Abstract

Strategies to investigate the possible existence of sporadic bovine spongiform encephalopathy (BSE) require systematic testing programs to identify cases in countries considered to have little or no risk of orally acquired disease or to detect a stable occurrence of atypical cases in countries in which orally acquired disease is disappearing. To achieve 95% statistical confidence that the prevalence for sporadic BSE is no greater than 1 per million (i.e., the annual incidence of sporadic Creutzfeldt-Jakob disease [CJD] in humans) would require negative tests in 3 million randomly selected older cattle. A link between BSE and sporadic CJD has been suggested on the basis of laboratory studies but is unsupported by epidemiologic observation. Such a link might yet be established by the discovery of a specific molecular marker or of particular combinations of trends over time of typical and atypical BSE and various subtypes of sporadic CJD, as their numbers are influenced by a continuation of current public health measures that exclude high-risk bovine tissues from the animal and human food chains.

## Abstract 2

TOC Summary: Atypical BSE is probably not sporadic and not related to sporadic Creutzfeldt-Jakob disease.

Bovine spongiform encephalopathy (BSE) was first recognized in 1986 in the United Kingdom and quickly reached epidemic proportions, affecting >30,000 cattle per year by 1992. Because of continuing exportation of both live cattle and meat and bone meal rendered from the carcasses of slaughtered cattle, the disease spread throughout most of Europe and a few non-European countries. By 2006, 20 years after its first appearance in the United Kingdom, the disease had been reported in an additional 24 countries (*1*).

Beginning toward the end of the 1980s in the United Kingdom, and in the 1990s in other countries, numerous regulations were enacted to minimize the entry of contaminated tissues into both the animal and human food chains and to eliminate the international spread of disease. These measures have been extraordinarily successful, to the extent that no new countries have been added to the list during the past year and the number of new cases has dramatically diminished in most countries in which BSE has appeared (the situation in some countries with insufficient surveillance remains unclear).

Although the origin of the epidemic is thought to have been caused by a species-crossing contamination by sheep scrapie during the course of rendering and recycling carcass meat and bone meal as cattle feed, an alternative hypothesis suggested an origin in a similarly recycled case of spontaneously occurring disease in cattle. The pros and cons of these competing ideas have been argued elsewhere (*2**,**3*), and neither will ever be convincingly proved or disproved. Thus, the phenomenon of spontaneous disease remained in limbo until the recent discovery of "atypical" strains of BSE reopened the question. In this article we consider the importance of atypical BSE within the overall concept of sporadic (spontaneous) disease and whether such cases, if they exist, could account for at least some cases of apparently sporadic Creutzfeldt-Jakob (CJD) in humans.

## Sporadic BSE

Obviously, the ideal country in which to examine the question of sporadic BSE would have a large national herd that was guaranteed never to have been exposed to environmental sources of infection. Such an ideal will never be realized. Until recently, the United States appeared to have at least approached the ideal by having a large national herd, an adequate testing program, and an apparently small risk for contamination by imported cattle or cattle feed. That position was made vulnerable in late 2003 by the discovery of a case of BSE imported from Canada and was eliminated altogether by the subsequent discovery of 2 indigenously infected animals in widely separate regions of the country. Although the 2 indigenous cases might represent sporadic disease, the continuing identification of cases in western Canada, coupled with a history of substantial numbers of cattle imported from Canada into the United States (both indigenous US animals had the same molecular "signature" as the most recent Canadian case), makes it difficult to ignore the possibility of undetected instances of feed contamination from imported cattle and recycled infectious carcasses.

At present, the 2 best countries in which to undertake testing programs would be Argentina and Australia; both have large national herds (≈50 million and 30 million animals, respectively), and both are considered to be free of orally acquired BSE infections, on the basis of importation history, nutritional practices, and adequacy of surveillance (*4*). Even in these countries, however, the discovery of a case of BSE could not be guaranteed to be spontaneous because of the widespread global distribution of potentially infected cattle and cattle feed and the vagaries of international trade: imperfect record keeping, lack of compliance, and just plain deception.

By way of illustration, an incident occurred many years ago that involved a particularly bulky shipment labeled as a pesticide. The large quantity seemed unusual to the customs inspector, who opened it and discovered that the shipment contained meat and bone meal destined to be spread on fields to inhibit grazing by deer, a serious agricultural pest. Thus, a study of sporadic BSE would only be truly convincing if no cases were identified.

Moreover, the criteria for answering the question of sporadic BSE are different than for orally acquired BSE. Most importantly, we do not know at what age sporadic cases of BSE might occur, but they are unlikely to be in the 3- to 5-year-old age group in which orally acquired BSE is most prevalent. If the age distribution of sporadic disease in cattle were to mimic that of sporadic CJD in humans, it would not peak until 14–20 years of age (the last third of the ≈20-year natural life span of a cow). Substantial numbers of such older cattle do not exist, and thus it may never be possible to state with assurance that spontaneous BSE does not occur.

Even if we accept this practical constraint, we can still take advantage of the fact that in many countries a proportion of the total slaughter population consists of breeding stock and dairy cows that are culled at >7 years of age, and animals that go directly to rendering plants or die "on farm" further increase this number. Argentina, for example, with a national herd of ≈50 million cattle, in 2005 recorded nearly 1.4 million deaths from slaughter and natural causes in animals >7 years (L. Mascitelli, pers. comm.).

**Figure 1:**
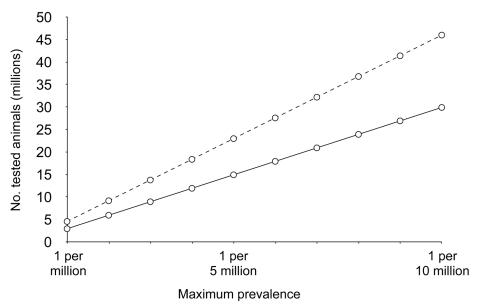
Distribution of ages at onset of illness in 500 cases of neuropathologically verified or experimentally transmitted sporadic Creutzfeldt-Jakob disease. Approximately 10% of cases occur in patients during the middle third (25–49 years) of a human lifespan, which corresponds to age in cattle of ≈7–13 years.

**Figure 2:**
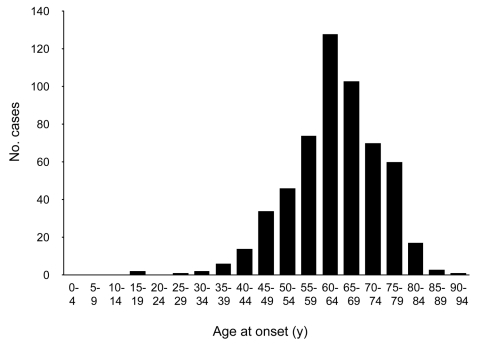
Maximum prevalence according to number of negative cattle at 95% (solid line) and 99% (dashed line) confidence levels. See Table 1 for exact numbers and statistical method.

Approximately 10% of cases of sporadic CJD occur in patients 25–50 years of age (Figure 1); this age in humans corresponds to the middle third of a cow's normal life span, or 7–13 years of age. If the age distribution of sporadic BSE followed the same pattern, negative test results in a total of ≈3 million animals randomly selected from this group would allow us to be 95% confident that sporadic BSE is not present at a prevalence >1 per million, and ≈4.5 million negative animals would raise the level of confidence to 99%. Larger numbers of BSE-negative animals would be required to achieve these levels of confidence for a maximum prevalence <1 per 10 million cattle (Table 1, Figure 2).

**Table 1:** Total number of older cattle with negative test results required to achieve 95% or 99% confidence* that sporadic cases of BSE are not present at a level higher than the illustrated prevalence rates†

Maximum prevalence	Log_10_ prevalence	No. tested cattle	
		95% Confidence	99% Confidence
1 per million	–6.000	2,995,731	4,605,168
1 per 2 million	–6.301	5,991,463	9,210,338
1 per 3 million	–6.477	8,987,195	13,815,508
1 per 4 million	–6.602	11,982,928	18,420,678
1 per 5 million	–6.699	14,978,660	23,025,849
1 per 6 million	–6.778	17,974,392	27,631,019
1 per 7 million	–6.845	20,970,124	32,236,189
1 per 8 million	–6.903	23,965,857	36,841,359
1 per 9 million	–6.954	26,961,589	41,446,529
1 per 10 million	–7.000	29,957,321	46,051,700

*α = 0.05 or 0.01. †The required number of tests, all of which must be negative, is given by log (α)/log (1-prevalence); BSE, bovine spongiform encephalopathy.

Even the least rigorous negative result—a prevalence not greater than that of sporadic CJD in humans, or 1 per million—would require several years to achieve, and it is perhaps unrealistic to suppose that the motivation to prolong the testing program will endure much beyond the global disappearance of orally acquired BSE and variant CJD. Nevertheless, to the degree that testing older as well as younger adult animals approached these numbers, both statistical and consumer confidence would increase, and at the very least provide reassurance that the occurrence of sporadic disease must be exceedingly rare, with little likelihood of posing a risk to either human or animal nutrition.

## Atypical BSE

Because of its contemporary nature, the study of atypical BSE is very much a work in progress, with comparatively little published data and many unknowns. The first 2 cases to be identified were a serendipitous discovery made in the course of an unrelated experimental study that required a detailed neuropathologic and immunochemical examination of the entire brain (*5*). The absence of clinical signs in these older animals, the unusual distribution of PrP^TSE^, together with amyloid plaques, and a Western blot pattern that differed from the stereotypic pattern seen in typical BSE left little doubt about the probability that a new "atypical strain" had been identified (bovine amyloidotic spongiform encephalopathy[BASE]).

**Figure 3:**
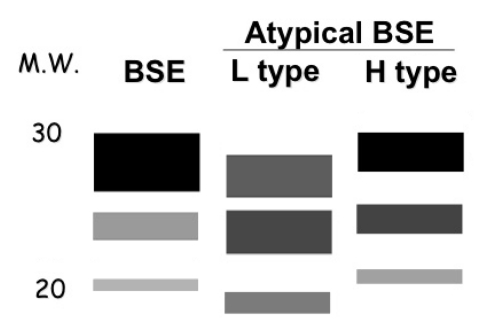
Representation of Western blots of PrP^TSE^ patterns of typical bovine spongiform encephalopathy (BSE) and the 2 major types of atypical BSE. M.W., molecular weight in kilodaltons; L type, atypical "light" pattern; H type, atypical "heavy" pattern.

Although no further cases were found in over 100 cattle examined in Italy, the initiation of Western blot studies of animals in other countries with screening test programs began to yield additional atypical patterns (Table 2, Figure 3) (6–14; P. Lind, pers. comm.). Two major patterns have been described, named L (resembling the original Italian case pattern with a lower molecular weight than typical BSE) and H (for a distinct pattern first seen in France with a higher molecular weight than typical BSE). It is not yet clear whether other mixed patterns result from technical procedures in different laboratories or whether a more complicated scheme of classification will evolve as more atypical patterns are discovered.

**Table 2:** Summary of atypical cases of bovine spongiform encephalopathy (BSE)

Country	Age, y	Breed	Symptoms	Neuropathology		Western blot pattern*
				Spongiform changes	Immunohistochemistry	
Italy	11	Bruna Alpina	None	Mild	Plaques	L
	15	Piemontese	None	Mild	Plaques	L
Denmark	14	Charolais	None	NR	NR	L
Poland	12	Black-white breed	None	Present	Positive (no plaques)	L
Japan	2	Holstein	None	Absent	Negative	L_1_
	14	Japanese Black	Dystasia	Severe	Positive (no plaques)	H
Belgium	5.5	East-Flemish	None	Absent	Negative	L_1_
France	10	Cross breed	None	NR	NR	H
	15	Prim Holstein	None	NR	NR	H
	8	Charolais	None	NR	NR	H
The Netherlands	13	Black-white Holstein**,** Freisian	NR	Present	No plaques	H
Sweden	12	Mixed Charolais	Recumbent	NR	Positive (no plaques)	H_1_
Switzerland	19	Zebu	Typical BSE	Typical BSE	Positive (no plaques)	H
Germany	13	Angus	NR	Absent	Positive (no plaques)	H
	15	Holstein-Freisian	NR	Absent	Positive (no plaques)	L
USA	12	Brahma cross	Falling	Absent	No plaques	H
	10	Red crossbred	Recumbent	Absent	No plaques	H
Canada	16	Charolais	Recumbent	NR	Positive (no plaques)	H

*L, lower molecular weight; H, higher molecular weight (the 2 major Western blot PrP glycopatterns that distinguish the strains from each other and from the pattern seen in typical BSE); NR, not reported. Only the Italian cows and Swiss zebu had full neuropathologic examinations (others were limited to examination of the obex). Details are not available for additional animals with both H and L strains in France and Poland.

In addition, Western blots of PrP^TSE^ are a fragile basis on which to define a BSE phenotype. Little or no information is available about either the clinical status or neuropathologic features of these animals. We know that cases have occurred in different breeds and PrP genotypes, and we also know that very few of the animals have had the typical clinical picture of BSE (behavioral disturbances, sensory signs, ataxia, and tremors), but a cloud of ambiguity surrounds the clinical picture even in those animals for which an extensive post-hoc investigation was undertaken. The fact that few detailed neuropathologic results are available is explained by the need to preserve at least a full half brain for examination, which is presently not done in any of the various countries that have screening test programs. In the future, the brain as well as the carcass must be retained in cold storage until the test results are known.

The frequency of atypical cases is another unknown. Published (*7**,**12*) and unpublished (*11**,**13*) observations indicate that in some countries it might be as high as 5%–10% of the total number of older animals diagnosed by rapid screening tests (e.g., 2/27 in Germany, and 1/9 in Canada), which would seem to be a surprisingly high proportion of spontaneously occurring cases. However, data are not yet sufficient to estimate the overall prevalence of atypical BSE, i.e., cases per million tested animals of all ages.

**Figure 4:**
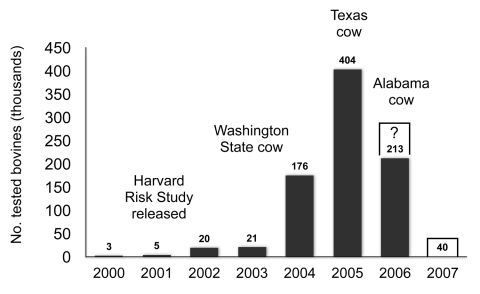
Numbers of tested cattle in the United States, 2000–2007. Number tested in 2006 as of August 20; number tested in 2007 proposed by the US Department of Agriculture.

In this context, a word is in order about the US testing program. After the discovery of the first (imported) cow in 2003, the magnitude of testing was much increased, reaching a level of >400,000 tests in 2005 (Figure 4). Neither of the 2 more recently indigenously infected older animals with nonspecific clinical features would have been detected without such testing, and neither would have been identified as atypical without confirmatory Western blots. Despite these facts, surveillance has now been decimated to 40,000 annual tests (USDA news release no. 0255.06, July 20, 2006) and invites the accusation that the United States will never know the true status of its involvement with BSE.

In short, a great deal of further work will need to be done before the phenotypic features and prevalence of atypical BSE are understood. More than a single strain may have been present from the beginning of the epidemic, but this possibility has been overlooked by virtue of the absence of widespread Western blot confirmatory testing of positive screening test results; or these new phenotypes may be found, at least in part, to result from infections at an older age by a typical BSE agent, rather than neonatal infections with new "strains" of BSE. Neither alternative has yet been investigated.

## Sporadic CJD

The possibility that at least some cases of apparently sporadic CJD might be due to infection by sporadic cases of BSE cannot be dismissed outright. Screening programs needed to identify sporadic BSE have yet to be implemented, and we know from already extant testing programs that at least a proportion of infected animals have no symptoms and thus would never be identified in the absence of systematic testing. Thus, sporadic BSE (or for that matter, sporadic disease in any mammalian species) might be occurring on a regular basis at perhaps the same annual frequency as sporadic CJD in humans, that is, in the range of 1 case per million animals.

Whether humans might be more susceptible to atypical forms of BSE cannot be answered at this time. Experimentally transmitted BASE shows shorter incubation periods than BSE in at least 1 breed of cattle, bovinized transgenic mice, and *Cynomolgus* monkeys (*12**,**13*). In humanized transgenic mice, BASE transmitted, whereas typical BSE did not transmit (*13*). Paradoxically, the other major phenotype (H) showed an unusually long incubation period in bovinized transgenic mice (*12*).

The limited experimental evidence bearing on a possible relationship between BSE and sporadic CJD is difficult to interpret. The original atypical BASE strain of BSE had a molecular protein signature very similar to that of 1 subtype (type 2 M/V) of sporadic CJD in humans (*5*). In another study, a strain of typical BSE injected into humanized mice encoding valine at codon 129 showed a glycopattern indistinguishable from the same subtype of sporadic CJD (*15*). In a third study, the glycopatterns of both the H and L strains of atypical BSE evidently did not resemble any of the known sporadic CJD subtypes (*12*).

To these molecular biology observations can be added the epidemiologic data accumulated during the past 30 years. The hypothesis that at least some cases of apparently sporadic CJD are due to unrecognized BSE infections cannot be formally refuted, but if correct, we might expect by now to have some epidemiologic evidence linking BSE to at least 1 cluster of apparently sporadic cases of CJD. Although only a few clusters have been found (and still fewer published), every proposed cluster that has been investigated has failed to show any common exposure to bovines. For that matter, no common exposure has been shown to any environmental vehicles of infection, including the consumption of foodstuffs from bovine, ovine, and porcine sources, the 3 livestock species known to be susceptible to transmissible spongiform encephalopathies. Additional negative evidence comes from several large case-control studies in which no statistically significant dietary differences were observed between patients with sporadic CJD and controls (*16**,**17*).

On the other hand, the difficulty of establishing a link between BSE and CJD may be compounded by our ignorance of the infectious parameters of a sporadic form of BSE (e.g., host range, tissue distribution of infectivity, route of transmission, minimum infectious dose for humans, whether single or multiple). Presumably, these parameters would resemble those of variant CJD; that is, high infectivity central nervous system and lymphoreticular tissues of an infected cow find their way into products consumed by humans. Transmissions that might have occurred in the past would be difficult to detect because meat products are generally not distributed in a way that results in detectable geographic clusters.

Barring the discovery of a specific molecular signature (as in variant CJD), the most convincing clue to an association will come from the observation of trends over time of the incidence of typical and atypical BSE and of sporadic and variant CJD. With 4 diseases, each of which could have increasing, unchanging, or decreasing trends, there could be 81 (3^4^) possible different combinations. However, it is highly likely that the trends for typical BSE and variant CJD will both decrease in parallel as feed bans continue to interrupt recycled contamination. The remaining combinations are thus reduced to 9 (3^2^), and some of them could be highly informative.

**Figure 5:**
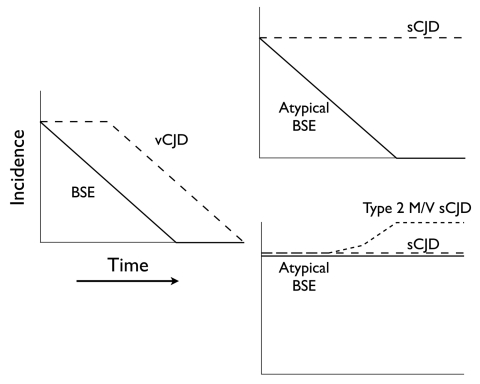
Diagram of 2 possible informative trends in the incidence of bovine spongiform encephalopathy (BSE) and Creutzfeld-Jakob disease (CJD). The left panel shows the likely trends of typical BSE and variant CJD (vCJD). The right upper panel shows 1 possible pair of trends of atypical BSE and sporadic CJD (sCJD) that might occur in conjunction with the typical BSE/vCJD trends, and would be consistent with the interpretation that atypical BSE is not sporadic and is not related to sCJD. The right lower panel shows a second possible associated pair of trends consistent with the interpretation that atypical BSE is sporadic and might also be related to the type 2 M/V subset of apparently sCJD.

For example, if the incidence of atypical BSE declines in parallel with that of typical BSE, its candidacy as a sporadic form of disease would be eliminated (because sporadic disease would not be influenced by current measures to prevent oral infection). If, on the other hand, atypical BSE continues to occur as typical BSE disappears, this would be a strong indication that it is indeed sporadic, and if in addition at least 1 form of what is presently considered as sporadic CJD (such as the type 2 M/V subtype shown to have a Western blot signature like BASE) were to increase, this would suggest (although not prove) a causal relationship (Figure 5).

Recognition of the different forms of BSE and CJD depends upon continuing systematic testing for both bovines and humans, but bovine testing will be vulnerable to heavy pressure from industry to dismantle the program as the commercial impact of declining BSE cases ceases to be an issue. Industry should be aware, however, of the implications of sporadic BSE. Its occurrence would necessitate the indefinite retention of all of the public health measures that exclude high-risk bovine tissues from the animal and human food chains, whereas its nonoccurrence would permit tissues that are now destroyed to be used as before, once orally acquired BSE has disappeared.
